# Reduction of Proliferating Olfactory Cells and Low Expression of Extracellular Matrix Genes Are Hallmarks of the Aged Olfactory Mucosa

**DOI:** 10.3389/fnagi.2018.00086

**Published:** 2018-03-27

**Authors:** Rumi Ueha, Shigeyuki Shichino, Satoshi Ueha, Kenji Kondo, Shu Kikuta, Hironobu Nishijima, Kouji Matsushima, Tatsuya Yamasoba

**Affiliations:** ^1^Department of Otolaryngology, The University of Tokyo, Tokyo, Japan; ^2^Department of Molecular Preventive Medicine, Graduate School of Medicine, The University of Tokyo, Tokyo, Japan

**Keywords:** olfactory receptor neurons, aging, extracellular matrix genes, interleukin-6, insulin-like growth factor 1

## Abstract

**Background:** The incidence of olfactory impairment increases with age; however, the detailed molecular and cellular mechanisms underlying this increase are yet to be determined.

**Methods:** We examined the influence of aging on olfactory receptor neurons (ORNs), which are maintained by a unique stem cell system, from olfactory progenitor cells to mature ORNs, by histological comparisons of the physiological status of the olfactory epithelium between young adult and aged mice. Furthermore, we clarified the expression of genes encoding inflammatory cytokines, neurotrophins, growth factors, and extracellular matrix proteins to reveal the molecular mechanisms underlying olfactory impairment caused by aging.

**Results:** The numbers of mature and immature ORNs, but not olfactory progenitors, decreased in the aged olfactory epithelium, with a concurrent reduction in Ki-67-positive proliferating cells. Transcriptome analyses revealed an increase in *Il6*, encoding a component of senescence-associated secretary phenotypes (SASP), and a decrease in *Igf1*, encoding a growth factor for ORNs, in the aged nasal mucosa. Interestingly, expression levels of several extracellular matrix genes, including *Col1a2*, decreased in the aged nasal mucosa. Consistent with the transcriptional changes, the number of *Col1a2*-GFP-positive cells decreased in the aged lamina propria.

**Conclusions:** Our data suggest that reduction in ORN number and cell proliferation, reduced extracellular matrix gene expression, and increased SASP contribute to olfactory impairment during aging.

## Introduction

Olfaction is impaired by a variety of factors, including environmental chemicals, upper respiratory tract infection, trauma, and, in particular, aging. Clinically, the elderly have a high incidence of olfactory impairment (Bihun and Percy, 1995) and post-viral olfactory dysfunction associated with the disruption of the olfactory neuroepithelium (olfactory epithelium: OE) (Moran et al., 1992; Yamagishi et al., 1994; Reden et al., 2006).

The olfactory system consists of peripheral compartments such as the olfactory mucosa including olfactory receptor neurons (ORNs), and central structures such as the olfactory bulb, the piriform/entorhinal cortex, anterior insula, orbitofrontal cortices, or the cortical nucleus of the amygdala. The olfactory mucosa is located in the posterosuperior nasal cavity, and the olfactory bulb is located at the rostral tip of the lower frontal lobe (Su et al., 2009). The olfactory mucosa consists of the OE and lamina propria. The OE of the olfactory mucosa has a unique regenerative stem cell system; it is maintained by the life-long replenishment of mature ORNs in the luminal layer from the stem/progenitor cells in the basal layer (Costanzo, 1991; Schwob, 2002; Su et al., 2009; Ueha et al., 2014). During steady-state neurogenesis, primitive olfactory progenitor cells differentiate into late progenitor cells, which subsequently differentiate into immature ORNs. Various stimuli, such as neurotrophins and cytokines, cause immature ORNs to differentiate into mature ORNs (Buiakova et al., 1996; Suzukawa et al., 2011; Nickell et al., 2012; Heron et al., 2013). Thus, progenitor basal cells have key roles in the olfactory system.

Aging-related olfactory dysfunction is associated with several histological changes in the OE, such as a thinning of the OE (Weiler and Farbman, 1997; Watanabe et al., 2006; Kondo et al., 2009) and respiratory epithelial metaplasia (Paik et al., 1992; Nagano et al., 1997; Rosli et al., 1999). In humans, part of the OE in the elderly is converted to a metaplastic respiratory epithelium, and the metaplastic regions increase with age (Holbrook et al., 2011; Suzukawa et al., 2011). In addition, there is a decrease in basal cell proliferation over time (Fung et al., 1997; Nagano et al., 1997; Watanabe et al., 2006). However, the detailed molecular mechanism underlying the aging-related disruption of the OE is yet to be determined.

In the steady state condition, neurotrophins and growth factors have important roles in the differentiation, maturation, and maintenance of neurons (Fernandez and Torres-Aleman, 2012; Liu et al., 2013; Ziegler et al., 2015). In inflammatory conditions, the production of these factors is regulated by inflammatory cytokines (tumor necrosis factor (TNF), interleukin (IL)-1β, and IL-6) (Gölz et al., 2006; Temporin et al., 2008). Recently, it has become evident that some of these inflammatory cytokines are expressed in senescent cells in the tissues of aged individuals, even in the absence of an external irritant. This senescence-associated secretory phenotype (SASP) has detrimental effects on tissue homeostasis. Considering previous reports, the balance of neurotrophins, growth factors, and cytokines might be important for aging-related dysregulation of OE homeostasis. Interventions to maintain the balance of these soluble factors could represent a novel treatment strategy against aging-induced olfactory impairment. However, the exact changes in conditions and regulatory mechanisms associated with aging remain unclear. Accordingly, in the present study, we contributed to clarify the effects of aging on the ORNs, from olfactory progenitor cells to mature ORNs, by histological comparisons of the physiological status of the OE between young adult and aged mice. Furthermore, we examined the expression of genes encoding inflammatory cytokines, neurotrophins, growth factors, and extracellular matrix proteins to reveal the molecular mechanisms underlying olfactory impairment caused by aging.

## Methods

### Mice

Two-month-old (young adult) and 16-month-old (aged) male C57BL/6 mice (*n* = 12 for each age group, 6 mice for histological analyses and 6 mice for gene analyses) were purchased from Saitama Experimental Animals (Saitama, Japan). Type I collagen-green fluorescent protein (Col-GFP) reporter mice (*n* = 5 for each age group) were provided by Y. Inagaki (Inagaki et al., 1994). Mice were housed in a temperature-controlled (23–25°C) environment under a 12-h light-dark cycle (light from 09:00 to 21:00 and darkness from 21:00 to 09:00 h) with access to food and water *ad libitum*. All animal experiments were conducted in accordance with institutional guidelines and with the approval of the Animal Care and Use Committee of the University of Tokyo (Approval No. P15-115).

### Tissue preparation

The septal nasal mucosa was harvested for histological and quantitative real-time polymerase chain reaction (qPCR) analyses, and the olfactory bulb was harvested for qPCR analysis. Immediately after the mice were sacrificed, the nasal cavities were gently irrigated with 4% paraformaldehyde to minimize mechanical damage to the OE. The mandibles were discarded and trimmed heads were skinned, fixed in 4% paraformaldehyde for 24 h, and decalcified for 7 days using Decalcifying Solution B (Wako Pure Chemical Industries, Ltd., Osaka, Japan). After decalcification, the tissues were dehydrated in a series of graded ethanol solutions and embedded in paraffin.

### Antibodies

Neurogenesis was examined following previously described methods (Buckland and Cunningham, 1999; Ueha et al., 2014, 2016a,b); tissues were stained with the following anti-mouse primary antibodies: SOX2 (1:300 dilution; rabbit monoclonal, Abcam clone EPR3131; Cambridge, UK), GAP43 (1:1,000 dilution; rabbit polyclonal, NOVUS #NB300-143B; Littleton, CO, USA), Ki-67 (1:200 dilution; rabbit monoclonal, Novus #NB600-1252), OMP (olfactory marker protein; 1:8,000 dilution, goat polyclonal, Wako), and cleaved caspase-3 (1:300 dilution; rabbit polyclonal, Cell Signaling #9661; Danvers, MA, USA). SRY (sex determining region Y)-box 2 (SOX2) is a transcription factor that is widely expressed in stem cell populations and is involved in maintaining the undifferentiated state. In the OE, SOX2 is expressed by proliferating stem cells or progenitor cells in the basal layer, and regulates homeostasis of the OE (Buckland and Cunningham, 1999; Ueha et al., 2014, 2016a,b). Growth associated protein 43 (GAP43) is expressed by immature ORNs in the OE (Ueha et al., 2016b). Ki-67 (antigen identified by monoclonal antibody Ki-67) is a cellular marker for proliferation, and Ki-67-positive cells are detected throughout the depth of the OE, particularly in the basal layer (Ueha et al., 2016a,b). Caspases are crucial mediators of programmed cell death (apoptosis), and caspase-3 (Cas3) is a frequently activated death protease, catalyzing the specific cleavage of many key cellular proteins (Ueha et al., 2016a,b).

### Histological analyses

**Figure 1:**
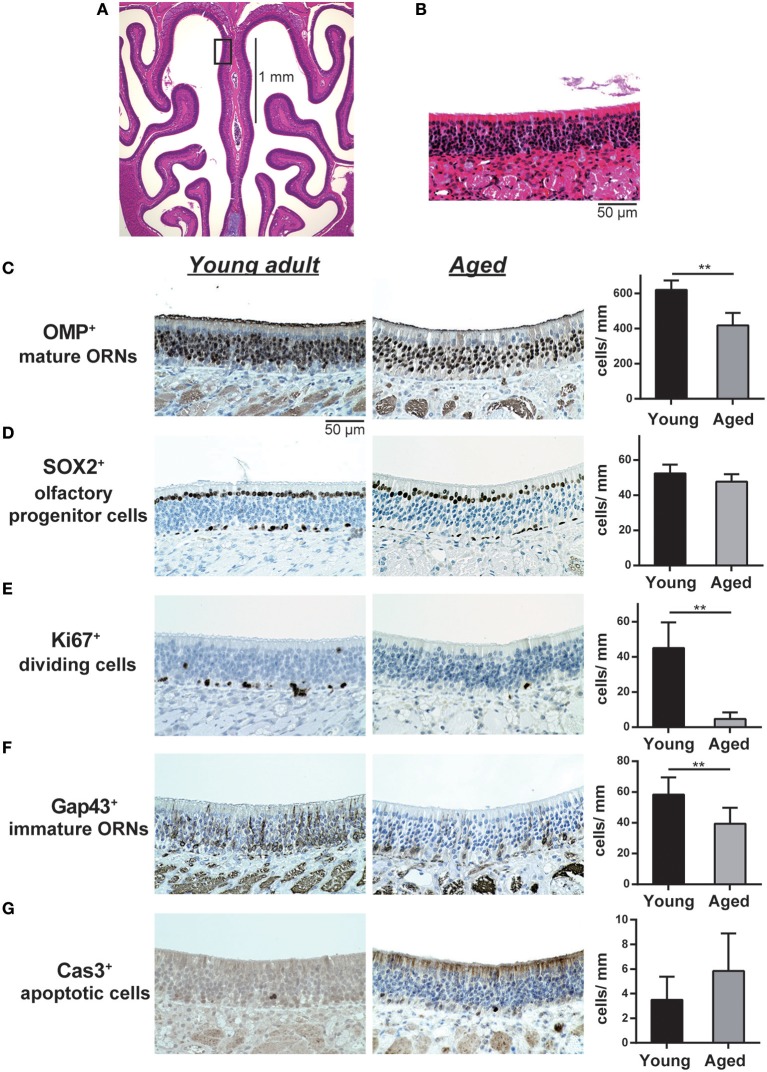
**(A,B)** Representative images of hematoxylin and eosin (H&E)-stained sections of the olfactory epithelium from young adult mice (**A**, 40× magnification; **B**, 400× magnification). A black line in **(A)** indicates the range for counting the number of each cell type. The box in **(A)** indicates the region of the olfactory epithelium shown at a representative higher magnification in **(B)**. Differences in the number of OMP^+^ mature olfactory receptor neurons (ORNs) **(C)**, SOX2^+^ ORN progenitors **(D)**, Ki-67^+^ proliferating cells **(E)**, GAP43^+^ immature ORNs **(F)**, and cleaved Cas3^+^ apoptotic cells **(G)** in the OE were evaluated by immunohistological staining (brown). Tissue sections were counterstained with the nuclear dye hematoxylin (blue). Representative images (400× magnification) of tissues stained with antibodies against olfactory marker protein (OMP), SRY (sex determining region Y)-box 2 (SOX2), Ki-67 (antigen identified by monoclonal antibody Ki-67), growth associated protein 43 (GAP43), and cleaved caspase 3 (CAS3) are shown. The number of cells per mm of basal layer length (**C–G**) was counted manually. Data represent the mean ± SD. ^**^P < 0.01 (*n* = 6, Mann–Whitney *U*-test).

All samples were cut at the level of the anterior end of the olfactory bulb as described previously (Ueha et al., 2014, 2016a,b). Four-micrometer-thick paraffin sections were deparaffinized in xylene, rehydrated in ethanol, and subjected to hematoxylin and eosin (H&E) staining or immunostaining. Before immunostaining, antigen-retrieval was performed using antigen retrieval solution (S1700; Dako, Tokyo, Japan) and sections were treated with 3% hydrogen peroxide to block endogenous peroxidase activity. The sections were then incubated with Blocking One (Nacalai Tesque, Tokyo, Japan) for 30 min at room temperature to block non-specific antibody binding. Primary antibodies were detected using peroxidase-conjugated secondary antibodies and a diaminobenzidine substrate. Images of bilateral septal OE were acquired using a digital microscope (BZ-X700; Keyence, Osaka, Japan) at 400× magnification (Figures 1A,B). Analyses were restricted to the OE of the nasal septum to minimize variation among specimens. The number of OMP^+^ ORNs, SOX2^+^ ORN progenitors, GAP43^+^ immature ORNs, Ki-67^+^ cells, and cleaved caspase-3^+^ (Cas3^+^) apoptotic cells per mm of basal layer length was counted manually using digital imaging software (Photoshop CS6; Adobe, San Jose, CA, USA) in a blinded manner. SOX2^+^ sustentacular cells in the luminal layer, which lack progenitor activity (Schwob, 2002), were excluded from our quantification of SOX2^+^ ORN progenitors. For analysis of GFP expression, decalcified tissues were placed sequentially in 10 and 30% sucrose solution for a few hours and embedded in Tissue-Tek Optimal Cutting Temperature compound (OCT compound) (Sakura, Tokyo, Japan). Six-micrometer-thick sagittal nasal sections were stained with DAPI (2-(4-amidinophenyl)-1H-indole-6-carboxamidine; Vector Laboratories, Burlingame, CA, USA), and fluorescence images were acquired using a BZ-X700 fluorescence microscope (Keyence, Osaka, Japan).

### Quantitative real-time polymerase chain reaction (qRT-PCR)

Total RNA was isolated from the septal nasal mucosae and olfactory bulbs using TRIzol reagent (Life Technologies, Tokyo, Japan) and converted to cDNA using ReverTra Ace qPCR RT Master Mix with gDNA Remover (Toyobo, Osaka, Japan), according to the manufacturer's instructions. qPCR was performed using the Thunderbird Probe qPCR Mix or Thunderbird SYBR qPCR Mix (Toyobo) and an ABI 7,500 real-time PCR system (Life Technologies). *Rps3* (encoding ribosomal protein S3) was used as an endogenous control (assay number Mm00656272_m1, Life Technologies). The expression levels of each gene were normalized against the expression level of *Rps3* in each sample. The gene-specific primers and probes are shown in Table 1.

**Table 1:** Primers and probes used for real-time PCR.

**Gene**		**Sequence (5′-3′)**	**Dye**
*Col1a1*	Forward	CGATGGATTCCCGTTCGAGT	FAM/TAMRA probe
	Reverse	GAGGCCTCGGTGGACATTAG	
	Probe	CCGACCCCGCCGATGTCGCTATCCAGCT	
*Col1a2*	Forward	GCTGGTGTAATGGGTCCTCC	
	Reverse	CGACCGGCATCTCCATTAGG	
	Probe	TGGCAATCGTGGTTCAACCGGCCCTGC	
*Col3a1*	Forward	GACCTAAGGGCGAAGATGGC	
	Reverse	GAAGCCACTAGGACCCCTTTC	
	Probe	TGGTGCAAATGGGCTTCCAGGAGCCGCA	
*Fn1*	Forward	CCCAGCTCACTGACCTAAGC	
	Reverse	TTCTCCTGCCGCAACTACTG	
	Probe	AAGCATCGGCCTGAGGTGGACCCCGCT	
*Col4a1*	Forward	CGCCTGGTACAAAAACCTCCA	SYBR green
	Reverse	CCGTGATAAAGTGCGTGCCA	
*Col5a1*	Probe	CTCAGAGCCCTCCCCCGCAACT	
	Forward	GCTGCTCACTGTGAAGGAAGT	
*Col6a1*	Forward	TGGGGAATGCATTTTACCAT	
	Reverse	AAATCGTGGTCCCCAAGC	
*Col10a1*	Forward	TCCTCATGTTTTGGGAACTATCT	
	Reverse	CGGTGCAGATTTCCAAGAAG	
*Tnf*	Forward	TGTGCCTCAGCCTCTTCTC	
	Reverse	GAGCCCATTTGGGAACTTCT	
*Il6*	Probe	CTGCAAGAGACTTCCATCCAGTT	
	Forward	AGGTCTGTTGGGAGTGGTATCC	
*Bdnf*	Forward	TCATACTTCGGTTGCATGAAGG	
	Reverse	AGACCTCTCGAACCTGCCC	
*Nt3*	Forward	GGAGTTTGCCGGAAGACTCTC	
	Reverse	GGGTGCTCTGGTAATTTTCCTTA	
*Gdnf*	Forward	CCAACTGGGGGTCTACGGA	
	Reverse	GCTTCGAGAAGCCTCTTACCG	
*Igf1*	Forward	AAAAGCAGCCCGCTCTA	
	Reverse	TCGATAGGGACGGGGACT	

### DNA microarray raw data collection

Total RNA was isolated from the nasal mucosa of young adult mice and aged mice using the TRIzol Reagent and RNeasy Mini Kits (QIAGEN, Venlo, Netherlands). RNA purity was confirmed based on the OD 260/280 ratio before analysis, using an Agilent 2,100 Bioanalyzer (Agilent Technologies, Santa Clara, CA, USA). Each total RNA sample was pooled from five biological replicates, yielding two pooled samples per experimental group. DNA microarray analyses were performed using the Agilent SurePrint G3 Mouse GE 8 × 60K Microarray (Agilent Technologies), according to the Agilent One-Color Microarray-Based Gene Expression Analysis protocol (Agilent Technologies, V 6.5, 2010) Raw data were extracted using the Agilent Feature Extraction Software (v11.0.1.1; Agilent Technologies). The raw data have been deposited in the NCBI Gene Expression Omnibus (https://www.ncbi.nlm.nih.gov/geo; accession GSE 103191).

### Microarray analysis

Background correction, averaging of the expression values of duplicated probes, and between-sample normalization of raw data were performed using Limma (Limma, 2005) and R 3.2.1 (https://cran.r-project.org/). Normalization was performed using the quantile method. The expression value for each gene was calculated by averaging the expression values of probes corresponding to each gene. The genes for which expression differences between young adult and aged mice were statistically significant were detected using Limma and R 3.2.1. Among these genes, those for which the mean normalized expression values across all experiments were <100 were filtered out, and those with a fold-change of ≥1.5 were identified as differentially expressed genes (DEGs) between young adult mice and aged mice.

### Gene ontology (GO) network analysis

DEGs were grouped into gene clusters with upregulated (C1, 390 genes) or downregulated (C2, 593 genes) expression in aged mice compared with young adult mice. A GO (Ashburner et al., 2000) network analysis for each DEG group was performed using Cytoscape version 3.3.0 (Bindea et al., 2009) with the ClueGO/CluePedia plugin (Bindea et al., 2009) as described previously, with some modifications (Shichino et al., 2015). Significantly enriched GO-biological process terms (Gene Ontology, 2015) (GO levels: 4–7) and Kyoto Encyclopedia of Genes and Genomes (KEGG) pathway terms (Kanehisa et al., 2016) were explored [minimum gene number = 6 (cluster C1) and 8 (cluster C2), percent minimum genes = 6% (cluster C1) and 8% (cluster C2)] and grouped, and a term-network was constructed based on the overlap of their elements (kappa score = 0.4). Leading terms within each group were defined as the most highly enriched term in each group. The GO term database and KEGG pathway term database were accessed on Sep 2, 2016 and Oct 2, 2016, respectively.

### Statistical analysis

Statistical comparisons between groups were performed using Mann–Whitney U tests implemented in GraphPad Prism (version 6.0; GraphPad Software, Inc., La Jolla, CA, USA). The z value and effect size (r) of Mann–Whitney U tests were calculated by using Microsoft R Open 3.4.2 (https://mran.microsoft.com/open). qPCR data were subjected to logarithmic transformation before analysis. *P* < 0.05 was considered statistically significant. In the microarray analysis, the eBayes function with Benjamini–Hochberg correction within the Limma package (Limma, 2005) was used to calculate the statistical significance and false discovery rate (FDR) of the expression difference of each detected gene between young adult and aged mice. FDR < 0.25 and *P* < 0.05 were considered statistically significant. During a subsequent GO network analysis, a two-sided hypergeometric test with Benjamini–Hochberg correction was performed using the ClueGO/CluePedia package and Cytoscape 3.3.0; GO/KEGG terms with *P* < 0.05 were considered significantly enriched.

## Results

### Reduction of mature and immature ORNs in the aged olfactory epithelium

We first investigated the influence of aging on the cellular composition of the OE by comparing the OE of 2-month-old young adult and 16-month-old aged mice. Primitive olfactory progenitors were identified based on SOX2 expression in the basal layer, and immature ORNs and mature ORNs were identified based on GAP43 and OMP expression in the OE, respectively. We found that the number of OMP^+^ mature ORNs was significantly lower in aged mice than in the young adult mice (*U* = 36, *z* = 3.066, effect size *r* = 0.885, *P* < 0.01). The number of GAP43^+^ immature ORNs also decreased in aged mice (*U* = 34, *z* = 2.566, effect size *r* = 0.740, *P* < 0.05), but there was no significant difference in the number of SOX2^+^ olfactory progenitors between aged and young adult mice (Figure 1).

We next analyzed the cellular mechanisms underlying the reduction of mature and immature ORNs in aged mice. The number of mature ORNs is determined by the balance between the local proliferation of their precursors and cell death; therefore, we analyzed the number of Ki-67^+^ proliferating cells and cleaved-caspase-3^+^ (cCas3^+^) apoptotic cells in the OE. Both Ki-67^+^ cells and cCas3^+^ cells were mainly detected in and proximal to the basal layer, where olfactory progenitors and immature ORNs give rise to differentiated progeny. The number of Ki-67^+^ cells was significantly lower in aged mice than in young adult mice (*U* = 36, *z* = 2.897, effect size *r* = 0.836, *P* < 0.01). No significant difference was observed in cCas3^+^ cells between aged and young adult mice, in part because of the insufficient number of cCas3^+^ cells in the OE in both groups for statistical analyses (Figure 1). Collectively, these results indicated that the number of immature and mature ORNs declines during aging, and this decline was partially explained by the reduced proliferation of olfactory progenitors or immature ORNs, rather than a reduction of the number of SOX2^+^ olfactory progenitors.

### Changes in gene expression in the olfactory mucosa during aging

The reduction of proliferating ORNs in the aged OE suggests that the microenvironment for the maintenance of ORNs is compromised in the aged OE. Therefore, we conducted a comprehensive microarray-based analysis of gene expression in the nasal mucosa to investigate the molecular mechanisms underlying aging-associated reduction of ORNs. We identified 389 significantly upregulated and 589 significantly downregulated genes, with expression differences of ≥150% between aged and young olfactory mucosa. The top 40 genes that were upregulated or downregulated with age are shown in Tables 2, 3, respectively. Among the upregulated genes, we detected the most prominent cytokine of the SASP, *Il6* (IL-6), which impairs the stemness of pluripotent progenitor cells (Al-Shanti and Stewart, 2012). No significant expression differences were detected for genes encoding major growth factors, such as *Bdnf* (BDNF, bone derived neurotrophic factor), *Gdnf* (GDNF, glial cell derived neurotrophic factor), *Nt3* (NT-3, neurotrophin 3), and *Nt5* (NT-5 neurotrophin 5), except that *Igf1* (IGF-1, insulin-like growth factor 1) expression decreased significantly. Interestingly, the expression levels of extracellular matrix organization-related genes, such as collagen genes (*Col1a1, Col2a1, Col3a1*, and *Col5a1*), were significantly lower in aged mice than in young adult mice. To determine the biological functions associated with age-associated differential gene expression, we conducted a functional analysis based on GO. The results revealed 24 biological functions associated with significant differential gene expression caused by aging (Table 4). Of these 24 biological functions, only two were associated with genes that were upregulated with age: olfactory transduction and steroid hormone biosynthesis. The remaining 22 biological functions were associated with genes that were downregulated with age (Table 4, highlighted in gray). In addition to the reduced expression of genes involved in the regulation of cellular responses to growth factor stimulation or responses to axon injury, and the reduced expression of transduction pathway-associated genes that regulate cell division or survival and neurogenesis via the PI3K-Akt signaling pathway (Al-Shanti and Stewart, 2012; Nieto-Estévez et al., 2016a), we also detected an active reduction in the expression of gene groups involved in cellular processes for extracellular matrix and collagen fibril organization and angiogenesis (Table 4).

**Table 2:** Genes that were upregulated with age.

**Symbol**	**Gene expression level**		***P*-value**	**AG/YA**
	**Aver of YA**	**Aver of AG**		
Prl	267.6	8679.4	1.16.E-06	32.4
Chil4	240.9	3484.9	2.62.E-07	14.5
8430422H06Rik	11.7	115.9	4.73.E-02	9.9
Gh	34.7	343.5	2.92.E-07	9.9
Chil3	778.0	7004.1	8.92.E-07	9.0
Syt10	169.2	1267.6	3.33.E-05	7.5
Serpina3n	1846.5	10528.9	1.05.E-06	5.7
E130116L18Rik	41.4	234.8	8.82.E-04	5.7
Moxd2	180.8	1015.9	4.38.E-05	5.6
Krtap11-1	264.3	1382.7	1.68.E-06	5.2
Serpina3j	125.7	640.7	4.42.E-06	5.1
Bpifb9b	8741.7	41730.2	6.00.E-06	4.8
Olfr533	67.6	288.4	1.13.E-04	4.3
Olfr1205	29.9	117.3	2.67.E-04	3.9
C130026I21Rik	175.8	674.0	4.03.E-06	3.8
Serpina3c	30.6	113.5	1.63.E-04	3.7
Gm10378	210.3	779.1	1.63.E-05	3.7
Olfr1508	148.9	534.4	3.40.E-06	3.6
Nppc	133.8	475.6	8.24.E-06	3.6
*Il6*	34.6	122.9	5.04.E-05	3.5
Olfr1201	29.0	101.9	1.50.E-03	3.5
Olfr212	139.4	473.2	1.36.E-04	3.4
Sprr2f	56.7	191.5	4.08.E-04	3.4
H2-M10.3	425.5	1427.3	1.11.E-05	3.4
Cga	27.6	91.2	4.83.E-03	3.3
Scgb1b2	45.3	148.8	9.17.E-04	3.3
Olfr5	128.9	417.8	3.55.E-05	3.2
Olfr325	29.0	92.5	1.08.E-04	3.2
Twist2	215.2	685.8	1.58.E-05	3.2
H2-M10.2	43.3	137.6	8.57.E-03	3.2
1700016D02Rik	87.1	273.1	1.12.E-04	3.1
Speer5-ps1	237.2	741.3	6.07.E-05	3.1
Sult6b1	623.2	1924.8	4.19.E-06	3.1
Klrb1c	77.9	228.1	2.66.E-02	2.9
Gm17019	86.8	253.5	1.96.E-05	2.9
Olfr222	202.9	570.2	1.05.E-04	2.8
Synpr	51.2	143.8	4.29.E-04	2.8
Gm10354	228.5	635.3	1.80.E-05	2.8
Olfr1360	58.2	159.5	1.25.E-04	2.7
Gm9948	86.6	231.8	3.26.E-03	2.7

*Aver, average; YA, young adult group; AG, aged group*.

**Table 3:** Genes that were downregulated with age.

**Symbol**	**Gene expression level**		***P*-value**	**AG/YA**
	**Aver of YA**	**Aver of AG**		
5430401F13Rik	60835.2	1864.4	1.24.E-07	0.031
LOC102638913	58452.3	1899.8	1.78.E-07	0.033
Apol10a	1299.8	194.8	4.26.E-06	0.150
Pip	10295.9	1951.4	1.56.E-05	0.190
A630073D07Rik	2475.9	492.5	7.84.E-06	0.199
Scgb1b20	19290.8	3903.6	1.01.E-05	0.202
Ttr	96.1	19.5	9.89.E-06	0.203
Scgb1b27	56207.6	12090.5	1.63.E-06	0.215
Muc5ac	2652.4	606.2	4.63.E-05	0.229
Col1a1	30967.0	7684.5	4.36.E-06	0.248
Gm16070	174.9	43.4	3.86.E-02	0.248
Gm5154	118.5	30.1	4.15.E-03	0.254
Scgb1b30	867.3	224.0	2.23.E-03	0.258
Scgb1b3	68684.0	17808.7	1.25.E-05	0.259
Col3a1	5068.1	1334.0	5.02.E-06	0.263
Col2a1	1045.5	283.4	3.37.E-05	0.271
Ostn	130.5	35.5	2.70.E-04	0.272
Csn3	2885.2	804.2	3.72.E-06	0.279
Chil1	8162.7	2466.0	3.61.E-06	0.302
Chit1	168.1	51.1	9.75.E-04	0.304
Krt76	85.1	26.2	3.89.E-03	0.308
Ren2	809.1	257.1	2.44.E-04	0.318
Acp5	42434.3	13611.5	5.93.E-06	0.321
LOC382202	78.1	26.1	2.34.E-02	0.334
Gm9789	195.8	65.7	2.90.E-04	0.336
Olfr39	702.5	239.1	1.06.E-04	0.340
Scgb2b27	27679.9	9422.6	4.44.E-05	0.340
LOC102643057	99.0	34.0	2.92.E-03	0.343
Olfr702	180.7	62.4	4.47.E-02	0.345
Olfr566	1141.9	395.4	2.22.E-05	0.346
Col5a1	3340.0	1177.5	2.38.E-05	0.353
Gm13539	4076.0	1451.6	1.73.E-05	0.356
Scgb2b15	2610.0	931.0	7.95.E-05	0.357
Crisp1	35652.8	12737.7	6.27.E-06	0.357
Dcpp3	1417.2	506.3	2.65.E-05	0.357
Dcpp2	2909.5	1049.8	2.43.E-05	0.361
Ripply1	401.5	145.6	2.22.E-05	0.362
Cd163l1	996.8	363.2	1.19.E-05	0.364
Gm9779	444.1	163.0	4.90.E-03	0.367
Olfr420	268.9	98.9	1.08.E-05	0.368
*Igf1*	1090.0	648.0	4.66.E-04	0.594

*YA, Young adult group; AG, Aged group*.

**Table 4:** Functional analysis of upregulated and downregulated gene ontology (GO) terms.

**GO Term**	***P*-value**
Olfactory transduction	5.6.E-27
Extracellular matrix organization	1.1.E-13
Collagen fibril organization	2.2.E-10
Angiogenesis	1.6.E-09
Osteoblast differentiation	3.1.E-09
PI3K-Akt signaling pathway	6.7.E-09
ECM-receptor interaction	6.7.E-09
Regulation of vasculature development	1.5.E-07
Regulation of cell-substrate adhesion	1.3.E-06
Regulation of cellular response to growth factor stimulus	6.2.E-06
Tissue homeostasis	1.4.E-05
Collagen metabolic process	5.7.E-05
Amoebiasis	8.5.E-05
Reactive oxygen species biosynthetic process	9.3.E-05
Leukocyte chemotaxis	2.7.E-04
Regulation of embryonic development	7.2.E-04
Steroid hormone biosynthesis	8.2.E-04
Endodermal cell differentiation	1.1.E-03
Rheumatoid arthritis	2.0.E-03
Protein heterooligomerization	5.5.E-03
Response to vitamin	5.6.E-03
Segmentation	8.0.E-03
Response to axon injury	9.4.E-03
Cardiocyte differentiation	2.1.E-02
Osteoclast differentiation	2.3.E-02

*Genes downregulated with age are highlighted in gray*.

### High *IL6* and low *Igf1* gene expression levels in the nasal mucosa and the olfactory bulb of aged mice

**Figure 2:**
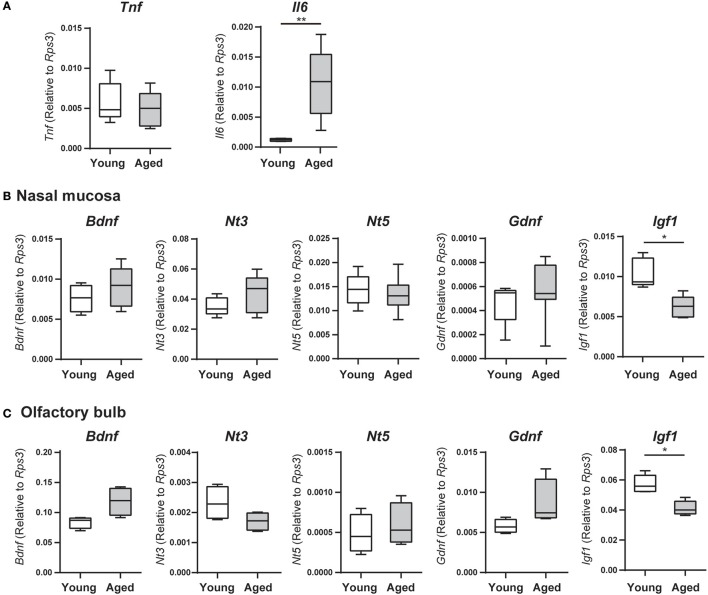
**(A)** Tumor necrosis factor (*Tnf*) and interleukin 6 (*Il6*) mRNA expression levels in the nasal mucosa were quantified by quantitative real-time PCR (qPCR) in young adult or aged mice, and are expressed relative to the expression level of the endogenous control gene *Rps3* (encoding ribosomal protein S3). Data represent the mean, minimum, and maximum (*n* = 6), and are representative of two independent experiments (^**^P < 0.01; Mann–Whitney *U*-test). **(B,C)** The nasal mucosae **(B)** and olfactory bulbs **(C)** of young adult and aged mice were collected for analysis of neurotrophin and growth factor mRNA expression by quantitative real-time PCR (qPCR), and expression levels are expressed relative to the expression of the endogenous control gene *Rps3* (encoding ribosomal protein S3). Data represent the mean, minimum, and maximum (*n* = 6), and are representative of two independent experiments (^*^P < 0.05; Mann–Whitney *U*-test).

We examined nasal mucosa mRNA expression levels of genes encoding the inflammatory cytokines TNF and IL6 by qRT-PCR. The expression levels of *Tnf* did not differ significantly between young adult and aged mice; however, *Il6* mRNA expression was significantly higher in aged mice than in young adult mice (*U* = 36, *z* = 3.066, effect size *r* = 0.885, *P* < 0.01; Figure 2A). We also examined the expression of genes encoding the neurotrophic factors BDNF, NT-3, NT-5, GDNF, and IGF-1, which are involved in neurogenesis of ORNs. In the nasal mucosa, the expression levels of *Bdnf*, *Nt3, Nt5*, and *Gdnf* mRNA did not differ significantly between young adult and aged mice, but *Igf1* mRNA expression was significantly lower in aged mice than in young adult mice (*U* = 25, *z* = 2.654, effect size *r* = 0.839, *P* < 0.05; Figure 2B). Similarly, in the olfactory bulb, only *Igf1* mRNA expression was significantly lower in aged mice than in young adult mice (*U* = 25, *z* = 2.654, effect size *r* = 0.839, *P* < 0.05); there were no significant differences in the expression levels of *Bdnf*, *Nt3, Nt5*, and *Gdnf* between the groups (Figure 2C).

### Decrease of extracellular matrix gene expression and type 1 collagen-producing cells in the nasal mucosa of aged mice

**Figure 3:**
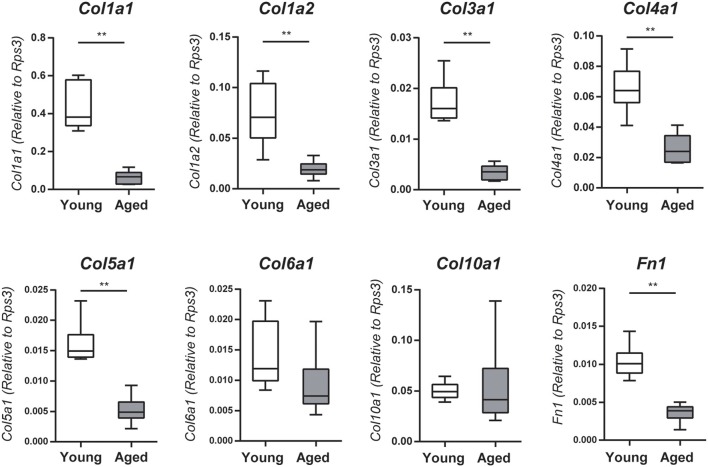
Collagen *(Col1a1, Col1a2, Col3a1, Col4a1*, and *Col5a1*) and fibronectin 1 (*Fn1*) mRNA expression levels in the nasal mucosa were quantified by quantitative real-time PCR (qPCR) in young adult or aged mice, and are expressed relative to the expression of the endogenous control gene *Rps3* (encoding ribosomal protein S3). Data represent the mean, minimum, and maximum (*n* = 6), and are representative of two independent experiments (^**^P < 0.01; Mann–Whitney *U*-test).

**Figure 4:**
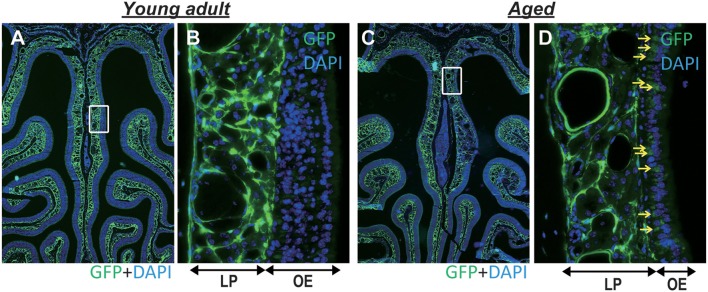
**(A,B)** Representative images of coronal sections of the olfactory epithelium from young adult type I collagen-green fluorescent protein (Col-GFP) reporter mice (**A**; 40× magnification, **B**; 400× magnification). **(C,D)** Representative images of the olfactory epithelium from aged mice. (**C**; 40× magnification, **D**; 400× magnification). The boxes in **(A,C)** indicate the region of the olfactory epithelium shown at a higher magnification in **(B,D)**. LP is the lamina propria, and OE is the olfactory epithelium. The density of Col-GFP-positive cells in the LP was lower in aged mice than in young adults. Yellow arrows indicate weak Col-GFP signals in the OE of aged mice.

The microarray and GO functional analyses demonstrated that a number of genes related to extracellular matrix organization and collagen fibril organization were downregulated with age; accordingly, we investigated mRNA expression levels of *Col1a1, Col1a2, Col3a1, Col4a1, Col5a1, Col6a1, Col10a1*, and *Fn1* by qRT-PCR. The gene expression levels of *Col1a1, Col1a2, Col3a1, Col4a1, Col5a1*, and *Fn1* (fibronectin 1) were significantly lower in the nasal mucosa of aged mice compared to young adult mice (*Col1a1*: *U* = 36, *z* = 3.066, effect size *r* = 0.885, *P* < 0.01; *Col1a2*: *U* = 35, *z* = 2.853, effect size *r* = 0.823, *P* < 0.01; *Col3a1*: *U* = 36, *z* = 3.066, effect size *r* = 0.885, *P* < 0.01; *Col4a1*: *U* = 35, *z* = 2.853, effect size *r* = 0.823, *P* < 0.01; *Col5a1*: *U* = 36, *z* = 3.066, effect size *r* = 0.885, *P* < 0.01; *Fn1*: *U* = 36, *z* = 3.066, effect size *r* = 0.885, *P* < 0.01; Figure 3). We also investigated the effects of aging on type I collagen-expressing cells using Col-GFP reporter mice. Col-GFP cells were mainly distributed in the lamina propria in both young adult and aged mice; however, the density of Col-GFP-positive cells was lower in aged mice than in young adults. Col-GFP-positive cells were rarely detected in the OE of young adult mice, whereas cells with weak Col-GFP signals were detected in the OE of aged mice (Figure 4). These results suggest that the quantity and quality of collagen-expressing mesenchymal cells change during aging.

## Discussion

Although the numbers of SOX2^+^ olfactory progenitor cells and cCas3^+^ apoptotic cells in the OE do not change during aging, our results demonstrated that the numbers of Ki-67^+^ dividing cells, GAP43^+^ immature ORNs, and OMP^+^ mature ORNs are reduced. The lack of a difference in the number of SOX2^+^ olfactory progenitor cells in the OE between aged mice and young adult mice suggested that the microenvironment of the aged OE preserves progenitor cells, but impairs their proliferation. Contradictory results have been obtained regarding changes in stem/progenitor cell populations caused by aging; some studies have reported age-dependent declines (Ryu et al., 2006; Watanabe et al., 2006; Tokalov et al., 2007; Aizawa et al., 2011), whereas others have indicated that there is no change resulting from aging (Sudo et al., 2000; Chang et al., 2007; Kim et al., 2008). Even in the OE, despite evidence that Musashi1^+^ stem/progenitor cells decrease due to aging (Watanabe et al., 2006), caution is needed when comparing those results with the results of the present study, because the marker used to detect stem/progenitor cells in the present study, SOX2, was different.

The expression levels of gene groups related to olfactory transduction in the OE and the inflammatory cytokine Il-6 were upregulated with age. Additionally, genes related to tissue structure, such as *Col1a1* and *Col1a2, Col3a1, Col4a1, Col5a1*, and *Fn1*, as well as the growth factor IGF-1, were significantly downregulated with age. The downregulation of genes related to tissue structure may reflect age-related atrophy of the entire nasal mucosa, or a decrease in tissue density. In addition, a histological analysis using Col-GFP mice revealed that type 1 collagen levels are decreased in the nasal mucosa of aged mice; thus, the quantity and quality of collagen-expressing mesenchymal cells might change with age. Despite clinical evidence that olfaction decreases with age, the mechanism underlying the upregulation of genes involved in olfactory transduction has yet to be definitively determined; however, it is speculated to be a compensatory response to olfactory impairment or an increase in the expression of genes in the nasal mucosa involved in olfactory transduction, such as *Syt10* (Cao et al., 2011), *Bpifb9b* (Kuntová et al., 2018), and *Srd5a2* (Steel and Hutchison, 1988).

Generally, in mammals, aging is accelerated by sustained mild inflammation because of the age-associated buildup of inflammatory cytokines (TNF, IL-1β, and IL-6) within tissues (Chaker et al., 2015). Under normal conditions, cells have mechanisms to prevent abnormal cell growth. One of these mechanisms is senescence, an irreversible phenomenon in which cells cease to divide, and this process increases with age (Maggio et al., 2006), giving rise to SASP. Inflammatory cytokines and chemokines, as well as extracellular matrix degradative enzymes, are secreted from cells that have undergone senescence (Hayflick and Moorhead, 1961; Jenny, 2012). This elevation in inflammatory cytokines results in a sustained state of chronic inflammation in aged animals. Indeed, the results of the present study indicated that the expression of the inflammatory cytokine IL-6 is increased in the nasal mucosal epithelium of aged mice, corroborating previous results for other tissues.

**Figure 5:**
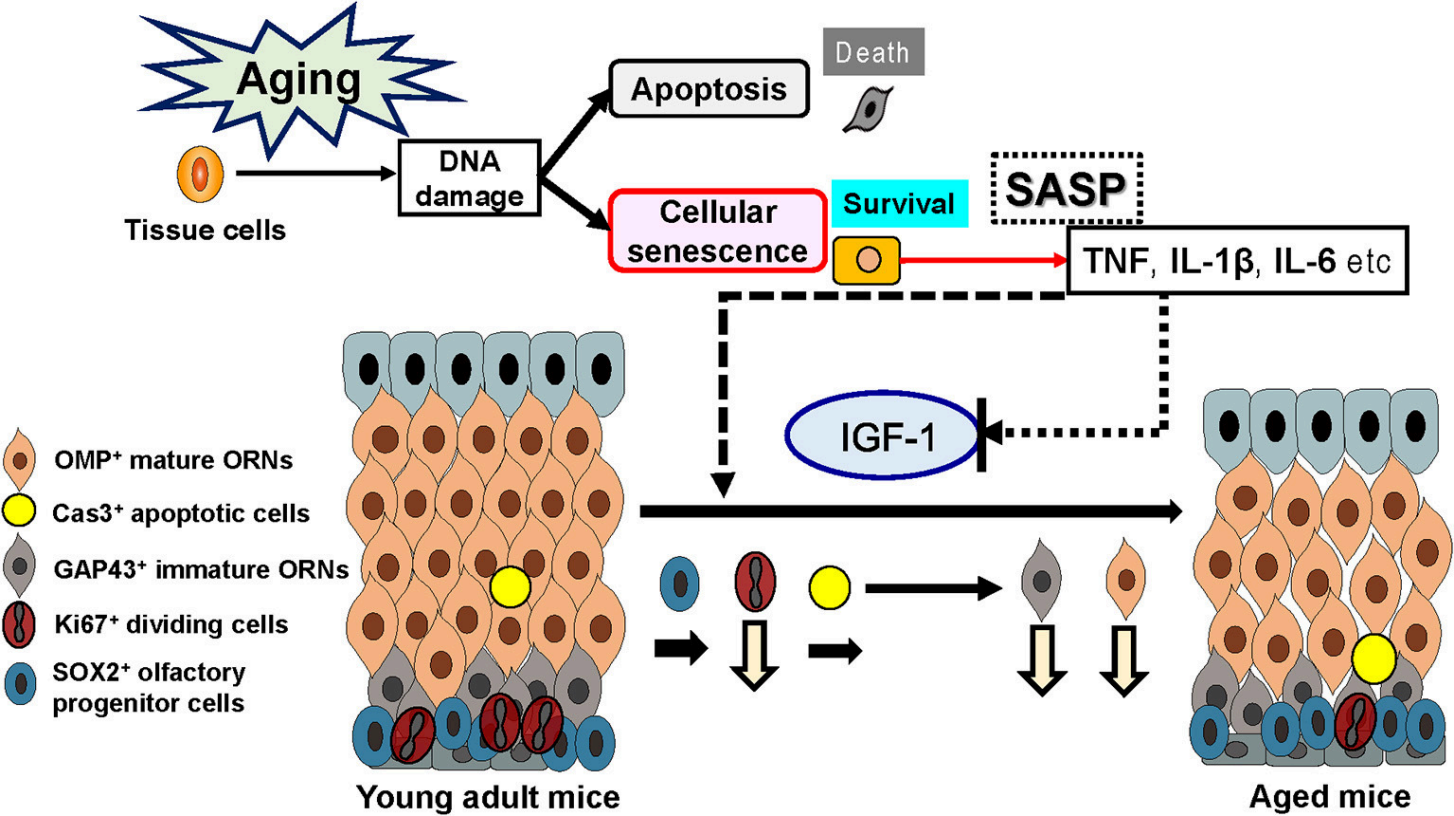
Effects of senescence-associated secretory phenotype (SASP) and insulin-like growth factor-1 (IGF-1) on the olfactory epithelium. Increased SASP may contribute to the aging-associated impairment of the olfactory stem cell system.

IGF-1 is a growth factor that promotes the genesis and growth of neurons and is mainly generated in the liver and skeletal muscle; however, it is also produced in the central nervous system, where it plays an important role in neuronal differentiation and maturation (Nishida et al., 2011; Oh et al., 2014; Byun et al., 2015; Nieto-Estévez et al., 2016a,b; Pardo et al., 2016). Inflammatory cytokines affect IGF-1-dependent neurogenesis mechanisms. For instance, TNF inhibits IGF-1-induced neurogenesis (Grounds et al., 2008), and IL-6 also inhibits neurogenesis and maintenance mechanisms by the inhibition of IGF-1-induced ERK1/2 and Akt pathways (Kim et al., 2004; Lebrun and Van Obberghen, 2008; Al-Shanti and Stewart, 2012). Presuming that neurogenesis is also regulated by a similar mechanism in the OE, an environment high in IL-6 and low in IGF-1, which is associated with aging, may inhibit IGF-1 signal transduction. Furthermore, in olfactory systems, this causes a decline in the growth and/or differentiation of mature ORNs from olfactory progenitor cells, thereby resulting in a reduced number of mature ORNS, which is predicted to lead to a decline in olfactory function (Figure 5). Based on studies indicating that IGF-1 administration in the auditory system improves hearing in individuals with hearing impairment (Nakagawa et al., 2014), and that IGF-1 stimulates collagen biosynthesis and helps to prevent skin aging (Takasao et al., 2012; Blackstock et al., 2014), IGF-1 is expected to directly affect the olfactory system or have indirect effects by stimulating the synthesis of collagen type 1.

In the olfactory system of adult mice, the kinetics of BDNF (Uranagase et al., 2012), GDNF (Buckland and Cunningham, 1999), NT-3 (Liu et al., 2013), and IGF-1 (Pixley et al., 1998; Ueha et al., 2016b) expression are related to the course of neurogenesis and regeneration. However, in the present study, despite the finding that the expression of neurotrophin genes involved in neurogenesis and differential growth (e.g., *BDNF, GDNF, NT-3*, and *NT-5*) in the nasal mucosa did not differ between aged mice and young adult mice, IGF-1 expression was significantly reduced, suggesting that a regulatory system centered around IGF-1 is crucial for the maintenance of the olfactory nervous system in aged mice.

The lamina propria consists of loose connective tissue together with extracellular matrix (ECM) collagen fibers, mesenchymal (or mesenchymal-like) stem cells, and olfactory ensheathing cells, which ensheath bundles of ORN axons that extend from the OE (Lindsay et al., 2010; Chen et al., 2014; Tanos et al., 2017). ECM is a key component of stem cell niches, which consist of ECM molecules such as collagen I and IV, laminin, and fibronectin (Schenke-Layland et al., 2011), and is involved in various aspects of stem cell behavior, thus having an impact on tissue homeostasis and regeneration (Gattazzo et al., 2014). Additionally, in the olfactory mucosa, the ECM is considered to be an important component for maintenance of ORN homeostasis (Lindsay et al., 2010). Mesenchymal (or mesenchymal-like) stem cells and olfactory ensheathing cells in the lamina propria have been reported to have the ability to promote regeneration in the olfactory nervous system (Richter and Roskams, 2008; Ge et al., 2016). Considering that the lamina propria of the nasal mucosa is important for the support and nutrition of the OE, and regulates epithelial assembly and turnover, the thinning of the OE in aged mice may be affected by decreases in the expression of type 1 collagen and reduced mesenchymal cells in the lamina propria. The decreased expression of type 1 collagen in the lamina propria following low expression of IGF-1 in the nasal mucosa may contribute to a decline in functions that support the OE and to decreased differentiation and maturation of olfactory progenitor cells.

Our findings demonstrated a decline in the division and differentiation of neuroprogenitor cells and a decline in the number of mature ORNs associated with the decline in olfactory function related to aging. The results also revealed that the mechanism underlying this decline involves the upregulation of inflammatory cytokines and a decrease in the expression of *Igf-1* and tissue structure-related genes. Future studies should focus on inflammatory cytokine inhibitors, IGF-1 promoters and supplements, and activators of IGF-1 signal transduction, which may be important clinical targets for preventing the decline in olfactory function associated with aging.

## Conclusions

The present study showed that the decline in mature ORNs accompanying aging, which influences olfaction, may be caused by a decrease in dividing cells and immature ORNs, and not by a decrease in olfactory progenitor cells. Furthermore, changes in the tissue structure and microenvironment, including *Il6* and *Igf1* expression, involved in the maintenance of olfactory neural tissue, may be related to olfactory impairment by aging.

## Author contributions

RU, SS, SU, KK, and TY developed the concept, designed and performed the experiments, and analyzed the data. SK and HN performed the experiments and analyzed the data. KM helped us to perform microarray analysis and gene ontology network analysis. All authors contributed to interpretation of the data and writing of the manuscript.

### Conflict of interest statement

The authors declare that the research was conducted in the absence of any commercial or financial relationships that could be construed as a potential conflict of interest.
